# Activation Energies of Heterogeneous Electrocatalysis: A Theoretical Perspective

**DOI:** 10.1021/acsmaterialsau.3c00071

**Published:** 2023-12-19

**Authors:** Zachary Levell, Yuanyue Liu

**Affiliations:** † Texas Materials Institute and Department of Mechanical Engineering, The University of Texas at Austin, Austin, Texas 78731, United States of America

**Keywords:** heterogeneous electrocatalysis, density functional theory, activation energy, solid−water interface, electrical double layer, constant electrode potential, electrolyte ion

## Abstract

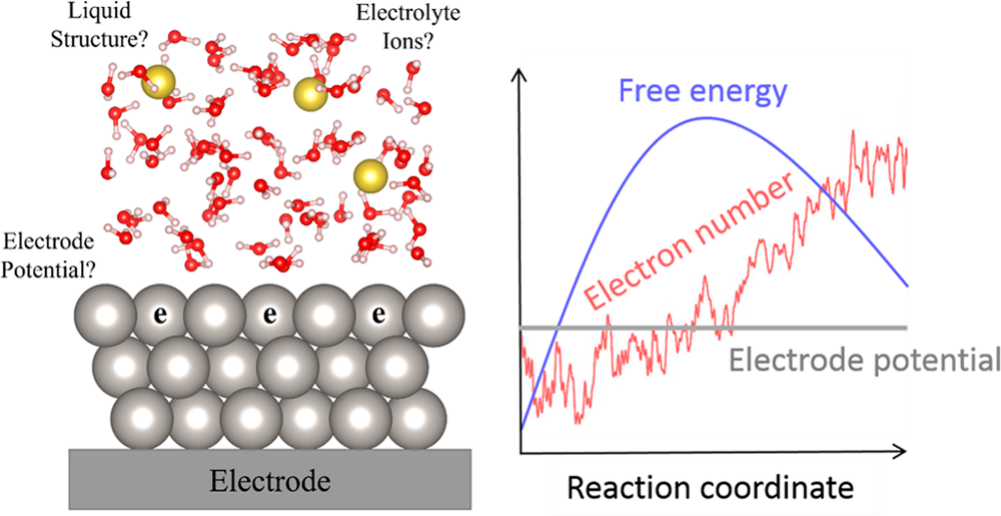

Heterogeneous electrochemistry is important for various applications. However, currently, there is limited information about activation energies. In this invited review, we review the challenges associated with calculating these activation energies. Specifically, we highlight three key difficulties in atomistic modeling: liquid structure, electrode potential, and electrolyte ions, along with state-of-the-art methods to address them. We aim to inspire more studies in the field of activation energies to better understand and design heterogeneous electrocatalysts.

## Introduction

Heterogeneous electrocatalysis is pivotal for a sustainable future. However, the existing catalysts generally suffer from low activity, selectivity, stability, and/or high cost, which necessitates a better understanding of the performance-limiting factors to facilitate rational design of new catalysts. To achieve this goal, it is important to develop computational tools for evaluating the catalyst performance from first principles.

Most studies that assess electrocatalyst performance are based on thermodynamics. Often, one calculates the free energy change for each elementary step along the reaction path and identifies the thermodynamic-limiting step. The catalyst with a less uphill (or more downhill) thermodynamic-limiting step is considered to be more active. However, this approach assumes that the thermodynamics are strongly correlated to the kinetics, which is not always true. Therefore, it is necessary to directly calculate the kinetics, especially the activation energy of each elementary step, to better understand and evaluate electrocatalysts.

## Discussions

**Figure 1:**
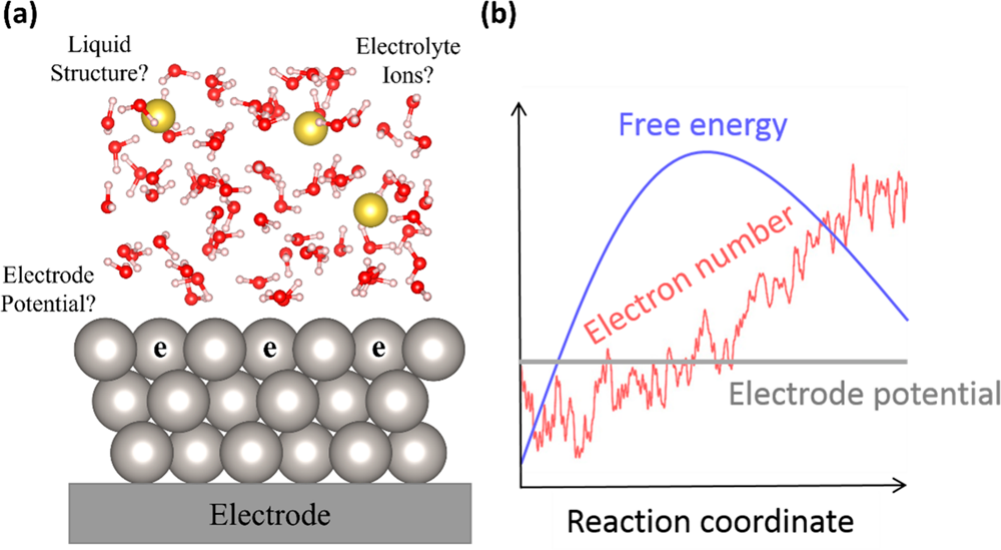
(a) Atomistic modeling of heterogeneous electrocatalysis is challenging due to “structure”, “electrode potential”, and “electrolyte ion” problems (see the main text), which hinder the calculations of activation energies. (b) Example evolutions of the free energy and electron number along the reaction coordinate during heterogeneous electrochemistry with a constant electrode potential.^1^ (b) is reproduced from ref (1). Copyright 2021 American Chemical Society.

Unfortunately, calculating kinetics is generally more difficult than thermodynamics. In addition to the general difficulty in searching for the transition state, there are other complexities specific to heterogeneous electrocatalysis. These challenges are summarized in Figure 1. First, heterogeneous electrocatalysis occurs at the interface between solid and liquid (often water). Simulating the amorphous and dynamic structures of liquids has been a long-standing challenge to the computational community. We call this the “*structure*” problem. Second, the catalyst can gain/lose electrons from/to the electrode to match its Fermi level (E_F_) with the electrode’s, which is defined by the externally applied electrode potential (U_ext_). This electron transfer will result in surface charges, which can significantly change the catalyst’s reactivity. Moreover, for an elementary reaction step, U_ext_ often remains constant (so-called “constant-potential” condition). This requires the number of electrons to change along the reaction path to maintain the equality between the catalyst’s and electrode’s E_F_, as depicted in Figure 1(b). This phenomenon is distinct from thermal reactions (which instead have a constant electron number), posing an additional challenge for atomistic modeling. We call this the “*electrode potential*” problem. Third, the surface charges are balanced by ionic charges in the solution, forming an electrical double layer. Describing the full atomic details of the ion distribution often requires a large model, as the ions usually extend deep into the solution. We call this the “*electrolyte ion*” problem. Note that these problems also exist for thermodynamic calculations but are less impactful.

At the simplest level, one can use the implicit solvation model to describe the liquid as a continuous medium.^2^ However, some important chemical interactions can be missing in this simplification. Ab initio molecular dynamics (AIMD) using explicit solvent molecules is a more accurate approach to model the liquid structure. When used together with enhanced sampling techniques (slow-growth,^3^ blue moon,^4,5^ metadynamics,^6^ etc.), one can obtain the free energy profile along the reaction coordinate and extract the activation energy. However, the high computational cost of AIMD limits the simulations to a short time (typically few picoseconds) and small size (usually hundreds of atoms), which is generally considered insufficient to sample the numerous solvent structures. To reduce the cost, the force field (FF) can be developed and used to replace the ab initio method for force calculation. The conventional approach to creating an FF is to manually construct a functional describing the interactions between atoms, which is quite difficult for complex systems. Machine learning (ML) emerges as an easier and universal approach to create FFs.^7^ With proper ML model design and training, FFs can have good accuracy at a much lower cost than the quantum mechanical method. ML FFs can similarly be combined with enhanced sampling to compute the activation energies. Note that current FFs do not consider the electrode potential effect and thus are not able to predict the potential dependence of the force as well as the charge dynamics. Integrating the electrode potential into the FF would be useful.

To account for the surface charge, it is necessary to know the number of electrons to be added/removed to/from the system for a given U_ext_. A straightforward approach is to tune the electron number so that the E_F_ reaches the target value set by the U_ext_. An efficient algorithm has been developed for the tuning.^8^ However, this does not accurately describe the electron statistics. A more realistic approach is to use dynamic equations of motion and electron number for a system under a thermostat and a potentiostat, the latter of which samples the grand-canonical distribution for electrons.^9,10^ In this case, E_F_ is not fixed; instead, it fluctuates around a constant set by U_ext_. In both cases, one needs to know the difference between E_F_ and −|e|U_ext_ for a given structure and electron number. Since U_ext_ is often referenced to the potential of standard hydrogen electrode (U_SHE_), the question becomes how to determine E_F_ vs SHE. A common solution is to create a region (vacuum or implicit solution) in the model where the electrostatic potential is uniform, which can then be used as a reference to compare the E_F_ of the system to that of SHE. Alternatively, one may use the computational standard hydrogen electrode (cSHE),^11,12^ which directly evaluates U_ext_ by determining the deprotonation energy of H_2_O/H_3_O^+^ at the interface.

The electrolyte ions can be modeled explicitly or implicitly. If represented explicitly, a common assumption is that they are concentrated on a plane above the surface to avoid the high cost of modeling the full distribution. Even so, one can only consider a discrete set of surface charges or U_ext_ because the number of explicit ions can only be an integer. Alternatively, they can be simplified to point charges as an implicit description. There are different fashions for placing the point charges. For example, they can be concentrated on a geometric plane,^13,14^ integrated into a plane of neon atoms,^15^ or spread uniformly in a region filled with implicit solvent.^16^ With a proper ionic distribution, one also obtains a region with uniform electrostatic potential that can be used to measure E_F_ relative to U_ext_. Note that different treatments of electrolyte ions will result in different capacitances, which will lead to different surface charges for a given U_ext_ and, consequently, different reactivities. It should also be noted that, similar to the electrons that can exchange with those in the electrode with a fixed potential, the electrolyte species in the reaction region can also exchange with those in the bulk solution with fixed chemical potentials. Therefore, in principle, they should also be treated using the grand-canonical ensemble. However, for fast reactions, the electrolyte species may not have enough time to exchange. In this case, it is reasonable to neglect the exchange.

To benchmark different methods, it would be ideal to compare computed activation energies to experimental data. Unfortunately, there is currently very little experimental information. Taking the simplest system, the hydrogen evolution reaction on a Pt(111) surface, as an example, there are few papers reporting the apparent activation energy in the past decades,^17−19^ and the reaction mechanism is unclear. Further challenges arise when considering more complex reactions and catalysts. Continued experimental efforts to resolve the activation energy and mechanism are highly desirable.

**Figure 2:**
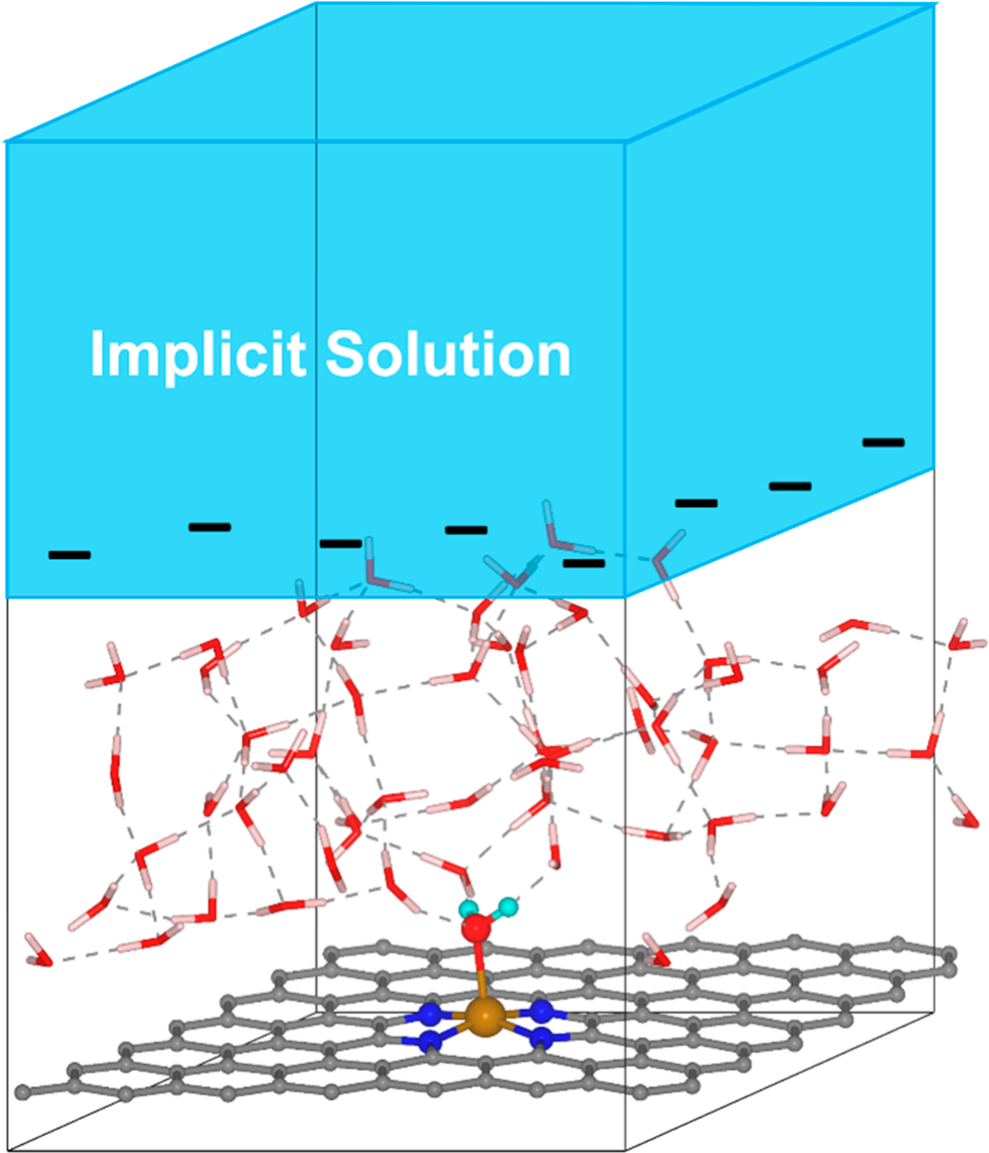
Illustration of the simulation cell for the CP-HS-DM, which is composed of an explicit region that contains the catalyst, reaction species, and some water molecules, as well as an implicit solution region with point charges that balance the extra electronic charges in the explicit region.

Although there are no well-established methods to address all these issues, cutting-edge methods have been applied to study the heterogeneous electrocatalysis. For example, Liu et al.^1^ used the constant-potential—hybrid solvation—dynamic model (CP-HS-DM) to answer the question how Co–N–C catalysts can produce H_2_O_2_ from oxygen reduction, despite being highly thermodynamically unfavorable compared with the H_2_O product. As shown in Figure 2, their system contains a thin film of explicit water molecules on top of the catalyst. The remaining space is filled with implicit solution containing point ionic charges distributed by the Poisson–Boltzmann equation, utilizing the VASPsol solvation model.^20^ They performed constant-potential AIMD simulation with the slow-growth method to calculate the activation energy.

To account for the surface charge, the electron number is adjusted every few MD steps according to the formula:
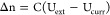
where Δn is the change in the number of electrons, U_curr_ is the potential of the system at the current MD step, and C represents the capacitance of the system, which is calculated as
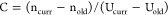
where n_curr_ is the number of electrons at the current MD step, n_old_ is the number of the electrons at the previous electron-adjusting step, and U_old_ is the potential at the previous electron-adjusting step. U is calculated as
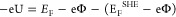
where E_F_ is the Fermi level of the system, Φ is the plane-averaged electrostatic potential in the implicit region far from the explicit region, and E_F_^SHE^ is the Fermi level of SHE. E_F_^SHE^ – eΦ is taken as −4.6 eV with the VASPsol solvation model and the PBE functional.^20^

**Figure 3:**
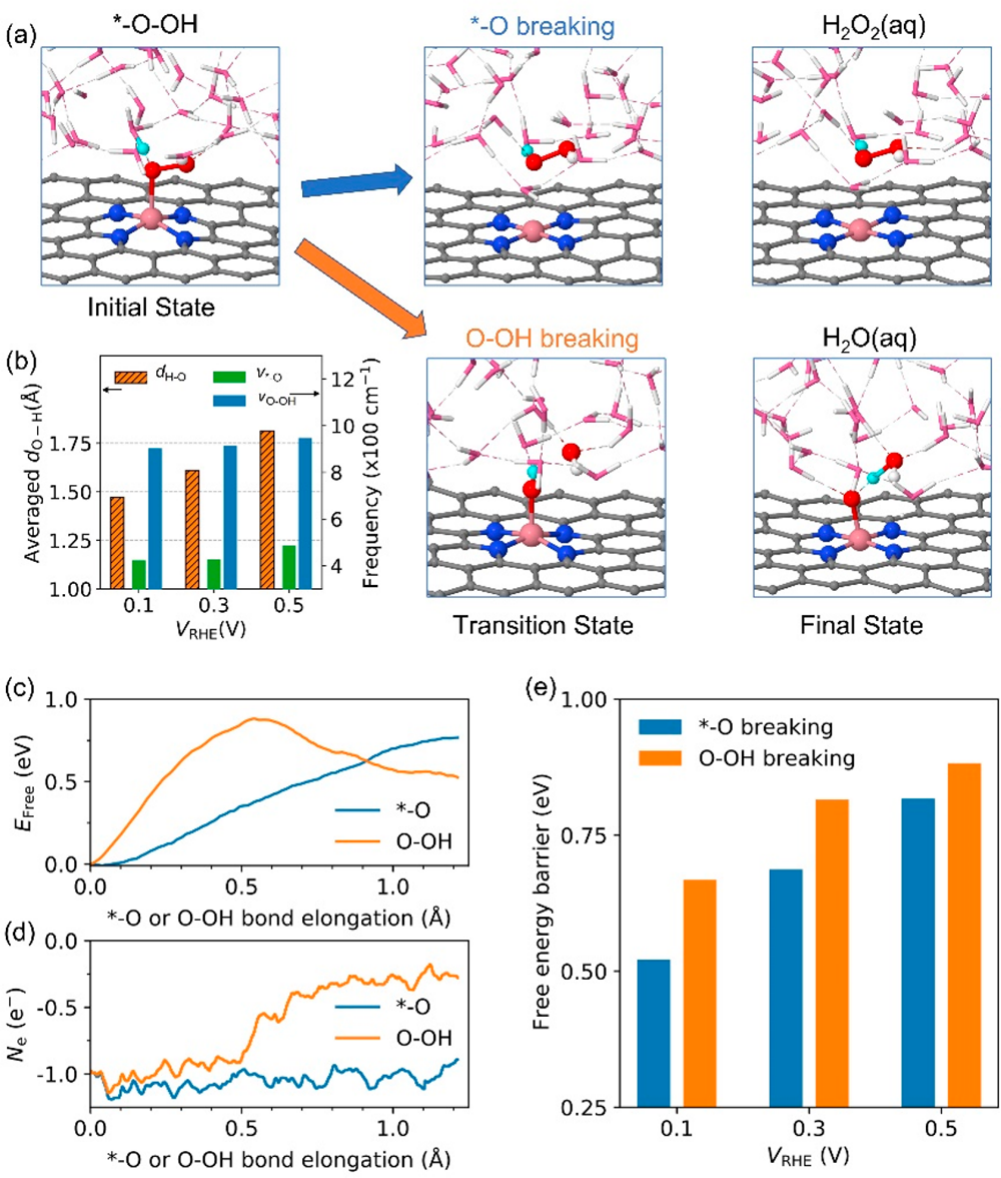
(a) Atomic structure evolution along two pathways of ORR. (b) Bond length and frequency vs electrode potential. (c) Free energy profile for breaking *–O bond and O–OH bond and (d) the corresponding electron number evolution. (e) Activation energies vs electrode potential. Reproduced from ref (1). Copyright 2021 American Chemical Society.

Figure 3(a) shows the structural evolutions for breaking the *–O bond to form H_2_O_2_ and for breaking the O–OH bond to form H_2_O. The corresponding energy evolutions are shown in Figure 3(c). They found that the activation energy to cleave the *–O bond is comparable to that for the O–OH bond, thus explaining the production of H_2_O_2_. Importantly, the system gains approximately one electron during H_2_O formation, whereas the charge is roughly constant during H_2_O_2_ (see Figure 3(d)), which is unobservable with constant-charge methods. Moreover, they found that decreasing U_ext_ enhances the H_2_O_2_ selectivity (see Figure 3(e)). This is because decreasing U_ext_ supplies additional electrons to the atom bound to Fe, which strengthens its attraction to nearby H_2_O and thus facilitates the H_2_O_2_ formation.

Although this work offers new insights into the kinetics of heterogeneous catalysis, improvements can be made to make the simulation more accurate, although with higher computational cost. For example, their slow-growth calculations are each performed for a few ps. The simulation time could be extended to check the convergence of the activation energies. Other enhanced sampling methods such as blue moon or metadynamics could be used to cross-check the barrier. Also, as mentioned above, a more accurate potentiostat can be used. Finally, the electrolyte ions are treated implicitly, which may overlook some important interactions between the ions and the reaction species. However, all of these improvements require higher computational cost, which is barely affordable by most researchers with current resources. Therefore, it is important to develop methods with a good balance between accuracy and efficiency.

Although no methods are perfect, we anticipate a growing number of calculations of the activation energies will significantly improve the understanding and design of heterogeneous electrocatalysts. As coined by George Box, “all models are wrong but some are useful”.

## References

[ref1] Zhao X.; Liu Y. Origin of selective production of hydrogen peroxide by electrochemical oxygen reduction. J. Am. Chem. Soc. 2021, 143 (25), 9423–9428. 10.1021/jacs.1c02186. 34133170

[ref2] Ringe S.; Hörmann N. G.; Oberhofer H.; Reuter K. Implicit Solvation Methods for Catalysis at Electrified Interfaces. Chem. Rev. 2022, 122 (12), 10777–10820. 10.1021/acs.chemrev.1c00675. 34928131 PMC9227731

[ref3] Jarzynski C. Nonequilibrium equality for free energy differences. Phys. Rev. Lett. 1997, 78 (14), 2690 10.1103/PhysRevLett.78.2690.

[ref4] Ciccotti G.; Ferrario M. Blue moon approach to rare events. Mol. Simul. 2004, 30 (11–12), 787–793. 10.1080/0892702042000270214.

[ref5] Sprik M.; Ciccotti G. Free energy from constrained molecular dynamics. J. Chem. Phys. 1998, 109 (18), 7737–7744. 10.1063/1.477419.

[ref6] Bussi G.; Laio A. Using metadynamics to explore complex free-energy landscapes. Nature Reviews Physics 2020, 2 (4), 200–212. 10.1038/s42254-020-0153-0.

[ref7] Unke O. T.; Chmiela S.; Sauceda H. E.; Gastegger M.; Poltavsky I.; Schütt K. T.; Tkatchenko A.; Müller K.-R. Machine Learning Force Fields. Chem. Rev. 2021, 121 (16), 10142–10186. 10.1021/acs.chemrev.0c01111. 33705118 PMC8391964

[ref8] Xia Z.; Xiao H. Grand Canonical Ensemble Modeling of Electrochemical Interfaces Made Simple. J. Chem. Theory Comput. 2023, 19 (15), 5168–5175. 10.1021/acs.jctc.3c00237. 37399292

[ref9] Bonnet N.; Morishita T.; Sugino O.; Otani M. First-Principles Molecular Dynamics at a Constant Electrode Potential. Phys. Rev. Lett. 2012, 109 (26), 266101 10.1103/PhysRevLett.109.266101. 23368585

[ref10] Deißenbeck F.; Wippermann S. Dielectric Properties of Nanoconfined Water from Ab Initio Thermopotentiostat Molecular Dynamics. J. Chem. Theory Comput. 2023, 19 (3), 1035–1043. 10.1021/acs.jctc.2c00959. 36705611 PMC9933428

[ref11] Cheng J.; Sprik M. Alignment of electronic energy levels at electrochemical interfaces. Phys. Chem. Chem. Phys. 2012, 14 (32), 11245–11267. 10.1039/c2cp41652b. 22806244

[ref12] Le J.; Iannuzzi M.; Cuesta A.; Cheng J. Determining Potentials of Zero Charge of Metal Electrodes versus the Standard Hydrogen Electrode from Density-Functional-Theory-Based Molecular Dynamics. Phys. Rev. Lett. 2017, 119 (1), 016801 10.1103/PhysRevLett.119.016801. 28731734

[ref13] Otani M.; Sugino O. First-principles calculations of charged surfaces and interfaces: A plane-wave nonrepeated slab approach. Phys. Rev. B 2006, 73 (11), 115407 10.1103/PhysRevB.73.115407.

[ref14] Hamada I.; Sugino O.; Bonnet N.; Otani M. Improved modeling of electrified interfaces using the effective screening medium method. Phys. Rev. B 2013, 88 (15), 155427 10.1103/PhysRevB.88.155427.

[ref15] Surendralal S.; Todorova M.; Finnis M. W.; Neugebauer J. First-Principles Approach to Model Electrochemical Reactions: Understanding the Fundamental Mechanisms behind Mg Corrosion. Phys. Rev. Lett. 2018, 120 (24), 246801 10.1103/PhysRevLett.120.246801. 29957006

[ref16] Kastlunger G.; Lindgren P.; Peterson A. A. Controlled-Potential Simulation of Elementary Electrochemical Reactions: Proton Discharge on Metal Surfaces. J. Phys. Chem. C 2018, 122 (24), 12771–12781. 10.1021/acs.jpcc.8b02465.

[ref17] Marković N. M.; Grgur B. N.; Ross P. N. Temperature-Dependent Hydrogen Electrochemistry on Platinum Low-Index Single-Crystal Surfaces in Acid Solutions. J. Phys. Chem. B 1997, 101 (27), 5405–5413. 10.1021/jp970930d.

[ref18] Schmidt T. J.; Ross P. N.; Markovic N. M. Temperature dependent surface electrochemistry on Pt single crystals in alkaline electrolytes: Part 2. The hydrogen evolution/oxidation reaction. J. Electroanal. Chem. 2002, 524–525, 252–260. 10.1016/S0022-0728(02)00683-6.

[ref19] He Z.-D.; Wei J.; Chen Y.-X.; Santos E.; Schmickler W. Hydrogen evolution at Pt(111) – activation energy, frequency factor and hydrogen repulsion. Electrochim. Acta 2017, 255, 391–395. 10.1016/j.electacta.2017.09.127.

[ref20] Mathew K.; Kolluru V. S. C.; Mula S.; Steinmann S. N.; Hennig R. G. Implicit self-consistent electrolyte model in plane-wave density-functional theory. J. Chem. Phys. 2019, 151 (23), 234101 10.1063/1.5132354. 31864239

